# Metagenomic Comparisons between Soft and Hard Feces of Plateau Pikas (*Ochotona curzoniae*)

**DOI:** 10.3390/ani12020149

**Published:** 2022-01-08

**Authors:** Haibo Fu, Wenjing Li

**Affiliations:** 1 Key Laboratory of Adaptation and Evolution of Plateau Biota, Northwest Institute of Plateau Biology, Chinese Academy of Sciences, Xining 810008, China; fuhb@nwipb.cas.cn; 2 Qinghai Provincial Key Laboratory of Animal Ecological Genomics, Xining 810008, China; 3 University of Chinese Academy of Sciences, Beijing 100049, China

**Keywords:** gut microbiota, plateau pika, soft feces, hard feces, cecotrophy

## Abstract

**Simple Summary:**

Plateau pika produces hard and soft feces with different morphology, component and microbial structure. Hard feces had more abundant Firmicutes, while soft feces had more abundant Akkermansia. The differences of microbial communities between hard and soft feces were mainly driven by core microbomes. Soft feces had a comprehensive advances in predict functional pathways compared to hard feces, these strengthened functional pathways were closely associated with metabolism of energy, vitamins, and amino acid. Our study preliminarily explored the differences in microbial structure and function between hard and soft feces, provided a foundation for future systematic explorations of the cecotrophy.

**Abstract:**

The division of hard and soft feces is an effective digestion strategy in the order Lagomorpha. Although previous studies have reported that hard and soft feces differ in morphology and component, the discrepancy in the microbiome remains unclear. This study explored the microbiomes of hard and soft feces in plateau pikas by sequencing the V3 and V4 regions of 16S rDNA. We found that hard feces harbored higher Firmicutes, while soft feces harbored higher *Akkermansia*. Increased rare bacterial taxa were observed in hard feces compared with soft feces. Moreover, hard and soft feces displayed a greater difference in terms of core operational taxonomy units (OTUs) compared to the total OTUs. The soft feces showed enhancements in all predicted Kyoto Encyclopedia of Genes and Genomes (KEGG) functions, indicating an advancing microbial metabolism compared to hard feces. The significantly upregulated pathways in soft feces were mainly enriched in metabolism of energy and carbohydrate, glycan biosynthesis, cofactors and vitamins, and amino acids—all of which are associated with increased contents of microbial proteins, vitamins, and short-chain fatty acids. Our study reports, for the first time, the differential microbiomes between hard and soft feces of pikas and provides direction for the future studies on cecotrophy.

## 1. Introduction

**Figure 1:**
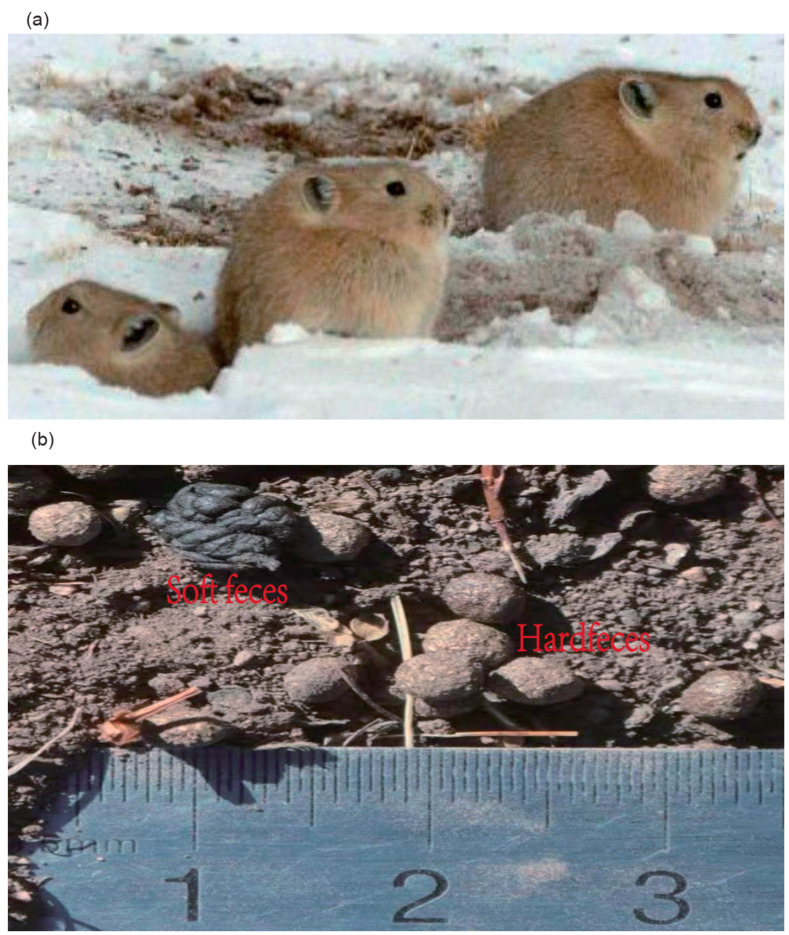
(**a**) plateau pikas (**b**) hard and soft feces.

Plateau pika is a keystone species for its widespread distribution and multiple roles in the alpine meadow ecosystem (Figure 1a) [1,2,3]. In this ecosystem, most mammalian and avian carnivores prey on pikas [1,2,3]. A pika’s burrow provides a habitat for endemic birds and reduces potential soil erosion caused by heavy rainfall [1,2,3]. Plateau pikas maintain plant species diversity through their behavior of digging [1,2,3]. Plateau pikas are the most widely distributed mammal on the Qinghai–Tibet Plateau (QTP) due to their excellent adaptability [4,5]. They maintain a high body temperature and a stable body mass in extremely cold and anoxic environments, without hibernation or food storage during winter [6,7]. Interestingly, the mortality of plateau pikas is lower in winter than in summer [8]. Despite several studies investigating their adaptive mechanisms, the available information remains limited. As a representative lagomorph, the body mass of plateau pikas is approximately 130 g [7]. The small body size restricts the ability to digest plants of low nutritional value [9]. However, pikas possess a unique digestive system and cecotrophy, thereby allowing them to consume low-quality forages [10]. Cecotrophy is a wise and successful strategy evolved from coprophagy, which contributes to the host having greater efficiency to recycle the energy and nutrition compared to coprophagy [10,11,12]. Cecotrophy is based on the colonic separation mechanism (CSM) at the proximal colon [11]; the pikas, thus, defecate not only conventional solid pellets (hard feces) but also soft dark feces, and they eat the soft ones by licking them from the anus when they are released (Figure 1b) [12,13]. These two types of feces differ in shape and component, formation mechanism, and microbial structure [14,15]. When producing hard feces, the fluid and fine food particles of the digesta are transported back to the cecum via the antiperistaltic movement of the proximal colon [16,17], and large particles are enriched in hard feces [18]. Thus, hard feces contain more poorly digestible particles. When producing soft feces, the fluid and fine food particles of the digesta are enriched in soft feces due to the reduction in and irregularity of the proximal colon’s mass peristaltic movement [17,18]. Therefore, soft feces are regarded as a representation of cecal contents with more proteins but less crude fiber than hard feces [10,19,20]. The CSM separates food particles, as well as microorganisms [14,21]. CSM and soft feces are closely associated with cecotrophy [20,22]. Preventing cecotrophy results in a decrease in body mass and an imbalance in host energy [23,24]. Therefore, cecotrophy in pika is an essential strategy for maximizing nutrient and energy absorption [25,26]. However, the microbiota differences between fecal types remains unclear.

The gut microbiome, which plays important roles in host energy harvesting, can be characterized using the fecal microbiome [27]. Although a previous study reported the influence of dietary factors on the microbiota of hard and soft feces in Lagomorpha [15], very little attention has been paid to the difference between fecal types based on the same dietary factor. Thus, this study aimed to determine the differences in the diversity, structure, and metabolic function of the gut microbiome between two types of feces in plateau pikas and to further investigate the implications of these differences.

## 2. Materials and Methods

### 2.1. Sample Collection

Adult plateau pikas were captured in Menyuan County of Haibei Tibetan Autonomous Prefecture, Qinghai Province, China (37.58° N 101.33° E, altitude: 3200 m) using a live-trapping way winter [6,7]. In total, we successfully fed 10 pikas and collected 10 pairs of fecal samples (Table S1). To ensure accuracy, the pikas were confined in cages sterilized regularly using 75% alcohol until they were excreted. Hard and soft feces were collected immediately following defecation. Samples were transferred to 2 mL tubes (DNase- and RNase-free; Eppendorf, Hamburg, Germany), followed by freezing with liquid nitrogen, and storage at −80 °C for subsequent analysis.

### 2.2. Sample Processing and 16S rDNA Amplicon Sequencing

We extracted the microbial DNA of feces using an Omega Biotek Stool DNA Kit D4015 (Omega Bio-tek, Inc., Norcross, GA, USA) [28,29]. Triplicate extractions were combined using QIAquick PCR Purification Kit (Qiagen, Valencia, CA, USA) and quantified using a Nanodrop ND-1000 Spectrophotometer (Nanodrop Technologies, Wilmington, DE, USA). PCR targeting the V3 and V4 regions of 16S rDNA was performed with the forward and reverse primers 341F (5′–CCTAYGGGRBGCASCAG–3′) and 806R (5′–GGACTACNNGGGTATCTAAT–3′) [28,29]. The PCR products were mixed in equal proportions of densities. Then, PCR products were purified using the SanPrep DNA Gel Extraction Kit (Sangon Biotech, Shanghai, China). To ensure no contamination, positive and negative controls were used during PCR. After equimolar pooling of PCR products, the resulting sequencing libraries were generated using the TruSeq DNA kit according to the manufacturer’s instructions (Illumina Inc., San Diego, CA, USA). The library quality was assessed on a Qubit 2.0 Fluorometer (Thermo Fisher Scientific, Waltham, MA, USA) and an Agilent Bioanalyzer 2100 system (Agilent Technologies Inc., Palo Alto, CA, USA). Paired-end DNA sequencing was performed using the HiSeq 2500 system (Illumina Inc., San Diego, CA, USA) in the laboratory of Genepioneer Biotechnologies Co. Ltd. (Nanjing, China).

### 2.3. Bioinformatics and Statistical Analysis

The paired-end reads were merged into one single tag on the basis of their overlapping regions using Fast-Length-Adjustment-of-Short-Reads version (FLASH) 1.2.11 [30], with a minimum overlap length of 10 bp and 2% mismatch allowed per overlapping region. Filtering operations were performed according to the protocols provided by QIIME pipelines version 1.9.1 [28,29,31]. Then, we aligned the clean tags against the Gold Database (r20110519) based on the UCHIME algorithm to identify and discard the chimers before obtaining the effective tags.

Before searching against the SILVA123 reference database, the effective tags were clustered into OTUs at a threshold of 97% identity by the uCLUST algorithm using VSEARCH v2.13.4_linux_x86_64 [28,29]. The representative OTUs were classified using PyNAST, and the taxonomy of the OTUs was assigned through the uCLUST algorithm [28,29].

The alpha and beta diversities were calculated using the script of alpha_rarefaction.py and beta_diversity.py in QIIME, and then they were visualized using GraphPad Prism v7.00 and R v3.2.2, respectively. PERMANOVA was run using the Adonis function based on 999 permutations in R v3.2.2. Linear discriminant analysis effect size (LEfSe) analysis was conducted using the Galaxy module (http://huttenhower.sph.harvard.edu/galaxy/, 8 January 2019). The Kyoto Encyclopedia of Genes and Genomes (KEGG) pathways were predicted using Tax4Fun v0.3.1 (http://tax4fun.gobics.de/, 28 December 2018) and annotated according to the KEGG database (https://www.kegg.jp/, 12 January 2019) using the K-number of OTUs. Significantly different pathways were extracted using White’s non-parametric *t*-test in STAMP v2.1.3. The core gut microbiota was defined as the OTUs that could be identified in all intra-group samples.

## 3. Results

### 3.1. Gut Microbiota Composition of Plateau Pikas

**Figure 2:**
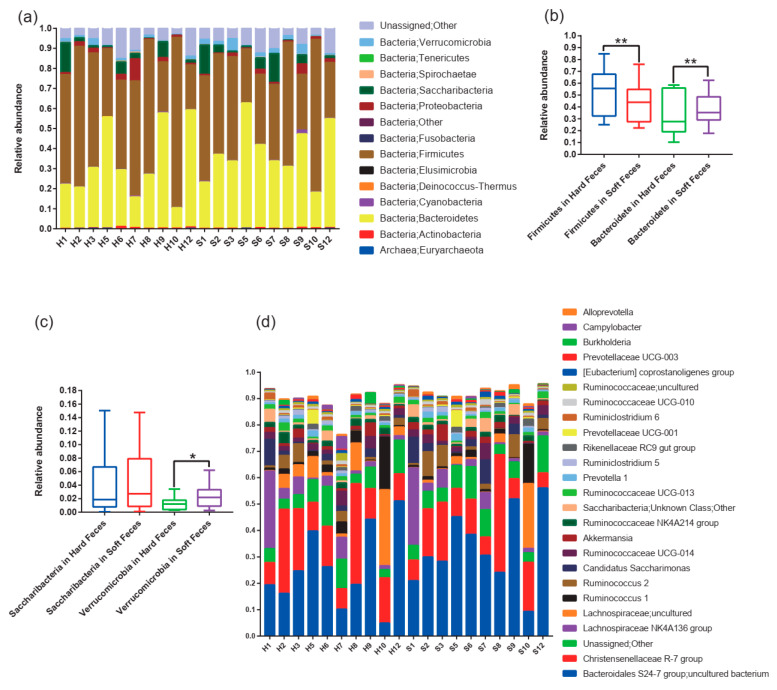
Composition of gut microbiota of hard feces and soft feces in plateau pikas. (**a**) Relative abundance of gut microbiota taxa at phylum level. (**b**,**c**) The dominant phyla; asterisks indicate the levels of statistical significance (Wilcoxon rank-sum test: ** *p* < 0.01, * *p* < 0.05). (**d**) The relative abundance of top 25 genera.

After filtering and sequence assembly, 1,993,407 valid 16S rDNA sequences from the 20 fecal samples were assigned to 1218 OTUs at a threshold of 97% identity. These OTUs spanned 15 phyla. Firmicutes, Bacteroidetes, Saccharibacteria, and Proteobacteria were the most dominant bacterial phyla in both hard and soft feces (Figure 2a), accounting for 51.6%, 32.5%, 3.8%, and 2.4% in hard feces, and 44.7%, 38.1%, 4.7%, and 1.6% in soft feces, respectively (Table S2). Furthermore, the relative abundance of Firmicutes was significantly higher in hard feces (51.6%) than in soft feces (44.7%) (*p* < 0.01) (Figure 2b). Conversely, Bacteroidetes (32.5% vs. 38.1%) and Verrucomicrobia (3.8% vs. 4.7%) showed significantly lower abundance in hard feces than in the soft feces (Figure 2b). No significant difference was observed in Saccharibacteria between fecal types (*p* > 0.05) (Figure 2c).

Genera from the Bacteroidales S24-7 group, Christensenellaceae R-7 group, and Lachnospiraceae NK4A136 group were the most abundant taxa in both types of samples (Figure 2d; Table S2). However, the relative abundances of the Christensenellaceae R-7 and Lachnospiraceae NK4A136 groups were almost identical between hard and soft feces, whereas the relative abundances of the Bacteroidales S24-7 group were 23.9% in hard feces and 34.0% in soft feces (Table S3).

### 3.2. Microbial Diversity of Pika

**Figure 3:**
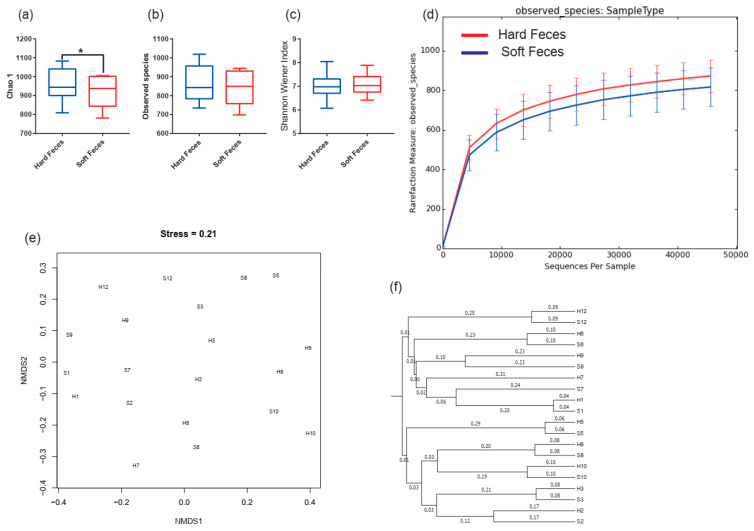
Alpha and beta diversity across all samples; asterisks indicate the levels of statistical significance between groups (Wilcoxon rank-sum test: * *p* < 0.05). (**a**) The Chao1 diversity index. (**b**) The observed species diversity index. (**c**) The Shannon–Wiener index. (**d**) Rarefaction curve. (**e**) Nonmetric multidimensional scaling (NMDS) based on Bray–Curtis distance. (**f**) Bray–Curtis dissimilarities cluster.

We calculated four metrics of the microbial alpha diversity indices: Chao1, observed species richness, Shannon–Wiener, and rarefaction curve (Figure 3a–d). When comparing these indices between fecal types, no significant differences were found except for a significantly higher Chao1 index in hard feces than in soft ones (Chao1: *p* < 0.05) (Figure 3a–d).

No significant divergence was observed between fecal types based on NMDS (Figure 3e) (PERMANOVA, Bray–Curtis metric: F_1,19_ = 0.06, R^2^ = 0.01, *p* = 0.987, permutations = 999). However, the hard and soft samples from the same pika were often clustered together and assigned to one clade in the Bray–Curtis dissimilarity (Figure 3e,f).

### 3.3. Core Microbial Community Better Reveals the Discrepancy between Hard and Soft Feces

**Figure 4:**
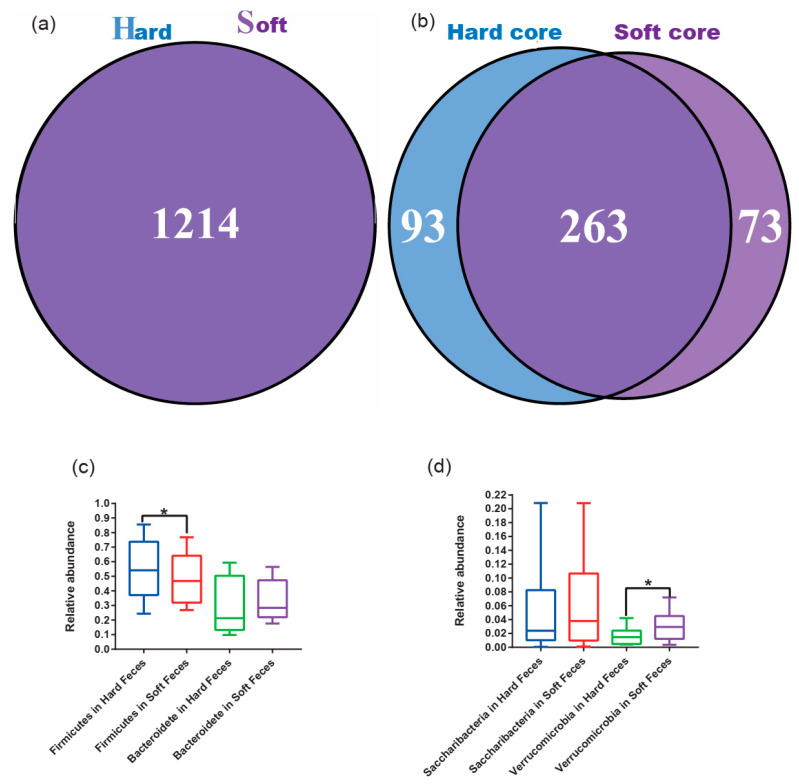
Core operational taxonomy units (OTUs) in hard (H) and soft feces (S); the “Hard core” and “Soft core” represent the core OTUs in hard and soft feces, respectively. (**a**) The shared OTUs between hard and soft feces based on the total OTUs. (**b**) The shared OTUs between hard and soft feces based on the core OTUs. (**c**,**d**) The dominant phyla (Wilcoxon rank-sum test: * *p* < 0.05).

No unique OTUs were identified in either fecal type on the basis of the total OTUs, suggesting that the two types of feces shared all OTUs (Figure 4a). This may have been caused by the horizontal transmission of microorganisms. Similar gut microbiotas have been observed in unrelated animals due to horizontal transmission via incidental contact [32]. The hard and soft feces pass through the same intestinal tract, resulting in inevitable contamination by horizontal transmission. Thus, excluding contaminable bacteria from the total gut microbes is necessary for exploring the actual structure of the gut microbiota. For this purpose, filtration from the total gut microbiota was performed to obtain the core microbes, as it reflects the stable colonizing bacteria in the host’s gut, and it is difficult to be disturbed by the surroundings [33]. Interestingly, a huge discrepancy, which was much more remarkable than the total gut microbiota, was observed in the core microbes between the hard and soft feces (Figure 4a,b). The hard and soft feces possessed 93 and 73 unique core OTUs, respectively, and they shared 263 OTUs (Figure 4b). The structure of the core microbiota was different at both the phylum and the genus levels (Figure S1a,b), and it was dominated by Firmicutes, Bacteroidetes, Saccharibacteria, and Verrucomicrobia at the phylum level (Figure S1a). Furthermore, a significantly higher abundance of Firmicutes was observed in hard feces than in the soft feces (*p* < 0.05), while a significantly lower abundance of Verrucomicrobia was identified in hard feces compared to the soft feces based on the core microbes (*p* < 0.05) (Figure 4c,d). At the genus level, there were also high-abundance bacterial genera in soft feces, including Propionibacteriaceae, *Propionibacterium*, Propionibacteriales, and Actinobacteria (Figure S1c).

### 3.4. The Predicted Function of Gut Microbial Communities in Hard and Soft Feces

**Figure 5:**
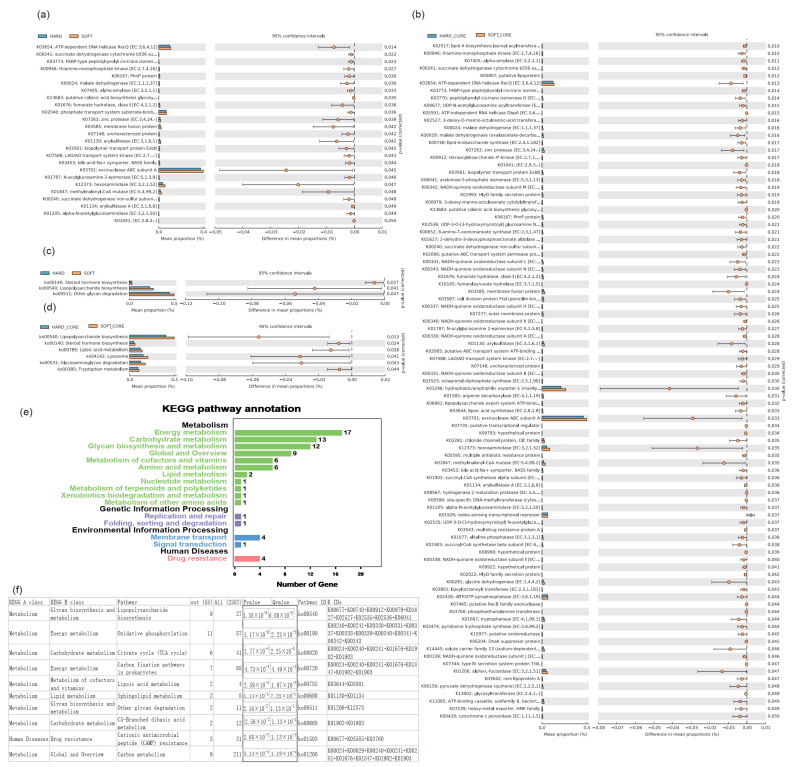
Significantly different KEGG functional pathways. The significance was measured in STAMP v2.1.3 using White’s nonparametric test (*p* < 0.05). (**a**) K-numbers based on the total OTUs. (**b**) K-numbers based on the core OTUs. (**c**) KEGG pathways based on the total OTUs. (**d**) KEGG pathways based on the core OTUs. (**e**) Annotated KEGG pathways based on the K-numbers of core OTUs. (**f**) Enrichment analysis based on the K-numbers of core OTUs (*p* < 0.05).

To explore the functional profile of gut microbiota from each fecal types, a functional prediction was made on the basis of the total OTUs. Interestingly, an extraordinarily consistent result was obtained, whereby all of the K-numbers with significant discrepancies had a higher abundance in soft feces than in hard feces (Figure 5a). Although the number of core OTUs was far less than the total (core: 429 vs. total: 1218) (Figure 4a,b), there were still 91 significantly different K-numbers identified from only 429 core OTUs (Figure 5b), whereas merely 25 significantly different K-numbers were identified from 1218 OTUs (total OTU number) (Figure 5a). The fact that fewer core OTU taxa yielded more functional differences suggests that the functional differences between fecal types were mainly driven by the core OTUs rather than the total OTU taxa. In addition, the 91 significantly different K-numbers consistently exhibited a higher relative abundance in soft feces compared to hard feces (Figure 5b), implying a more active bacterial metabolism in soft feces than in hard feces.

According to the total OTUs, the significantly different K-numbers were enriched in pathways associated with steroid hormone biosynthesis, lipopolysaccharide biosynthesis, and other glycan degradation (Figure 5c). Conversely, the significantly different K-numbers according to the core OTUs were enriched in lipopolysaccharide biosynthesis, steroid hormone biosynthesis, lipoic acid metabolism, lysosome and glycosaminoglycan degradation, and tryptophan metabolism (Figure 5d).

### 3.5. KEGG Pathway Annotation and Enrichment Analysis

To explore the enrichment profile of the differential genes, the K-numbers from core OTUs were used as background genes, and the K-numbers with significant differences were used as differential genes. Our analysis revealed that 80 significantly upregulated K-numbers in soft feces were further enriched in 16 pathways (Figure 5e,f). Most of the enriched pathways were closely associated with metabolism, especially the pathways of energy metabolism, carbohydrate metabolism, and glycan biosynthesis and metabolism, which harbored 17, 13, and 12 K-numbers, respectively (Figure 5e,f; Figure S2b,e; Figure S2a,c; Figure S3a,d). In addition, the pathways involved in important metabolism, such as global and overview pathways, metabolism of cofactors and vitamins, amino-acid metabolism, and lipid metabolism, were also enriched at a high K-number (Figure 5e,f; Figure S2d; Figure S3c; Figure S3b).

## 4. Discussion

Hard feces often contain largely indigestible particles of food digesta, which are composed of indigestible cellulose and hemicellulose [18]. Firmicutes plays an important role in fiber degradation and is closely associated with indigestible substances [34]. Increased Firmicutes was observed in hard feces (Figure 2b; Figure 4c). This may be due to the CSM, which combines indigestible particles and Firmicutes in hard feces, since the CSM separates not only the food digesta, but also microorganisms [12,21]. Differential distribution of microorganisms following the component disparity between fecal types may contribute the microorganisms to develop their own special skill to decompose the substrates as thoroughly as possible.

Most of the Verrucomicrobia in our study belong to *Akkermansia* (Table S1), which could stimulate the colon to produce more mucosa; subsequently, these mucosae can be used as the material of tough mucous membrane for soft feces [35,36]. Thus, enrichment of Verrucomicrobia (Figure 2c; Figure 4d) may have contributed to the formation of soft feces, as the soft feces were wrapped in mucosa [12]. Coupling with the movement of the colon, the mucosal membrane enriches the fine particle of digesta and microorganisms rich in bacterial protein-like pockets, and it subsequently packs them together in the soft feces [12,13]. After excretion, the soft feces are ingested in a batch without mastication by the hosts [37]. Hence, the tough mucous membrane protects the microorganisms inside the soft feces from the highly acidic environment of the stomach, allowing them to constantly degrade carbohydrates and produce SCFAs [38]. Therefore, the enrichment of Verrucomicrobia (*Akkermansia*) may play a fundamental role in the formation of soft feces and subsequent ingestion.

High dietary cellulose or hemicellulose often increases gut microbial diversity, especially rare bacterial taxa [39,40]. Hard feces, which contain more indigestible substrates, such as cellulose and hemicellulose, may provide an appropriate niche for the rare taxa and give rise to the taxon increasing [10]. Accordingly, a higher Chao1 and rarefaction curve may be driven by the cellulose and hemicellulose in hard feces (Figure 3a,d). Although hard and soft feces from the same pika were different, their microbial communities still clustered in one clade in Bray–Curtis dissimilarity (Figure 3f), suggesting that the host phylogeny is the most important shaper of gut microbiota, regardless of fecal type.

The core gut microbiota is often considered as the OTUs that were present in 100% of intra-group samples [41]; thus, it reflects the stable colonizing bacteria in the host’s gut, regardless of the environmental changes [33]. Upon formation, hard and soft feces are excreted at different time periods but pass through the same segment of the colon [42], resulting in contamination by residual microbes on the internal surfaces of the colon or microbes within the digesta. Therefore, microbial horizontal transmission would obscure the original distinctions, resulting in more shared OTUs between fecal types compared to the core OTUs (Figure 4a,b). Thus, the gut microbial composition in soft and hard feces may differ when initially produced and become increasingly similar as they pass through the same colon. Accordingly, unlike using the total microbiota, using the core microbiota may be an effective way to exhibit the different metagenomic characteristics between hard and soft feces.

In rabbits, soft feces harbor 30% microbial proteins and are twice as rich in protein as their vegetable diet [19]. Even in *Lepus*, soft feces are six times as rich in protein as the original diet (39.4% protein in soft feces), while hard feces only contained 8.7% crude protein [43]. This implies that an enrichment of nutrients was implemented during soft feces formation; meanwhile, high crude proteins in soft feces were caused by microbial nitrogen fixation rather than the original vegetable diet [25,43,44]. These studies may explain why all predicted KEGG functional pathways from soft feces were consistently enhanced compared to hard feces, but no KEGG functional pathway was more abundant in hard feces (Figure 5a–d). Thus, microbial nitrogen fixation underlies cecotrophy in lagomorphs, thereby enriching additional proteins for soft feces and providing ecological advantages for pikas to consume low-quality forages in harsh environments [13]. In our study, the significantly upregulated pathways in soft feces were mainly enriched in energy metabolism, carbohydrate metabolism, glycan biosynthesis and metabolism, metabolism of cofactors and vitamins, and amino-acid metabolism (Figure 5e,f). These results are consistent with previous studies showing that soft feces are richer in microbial proteins, vitamins, SCFAs, and the concentration of gut microorganisms than hard feces, all of which are crucial for host energy harvesting [10,38]. As a result, cecotrophy is an important adaptive mechanism in pikas; microorganisms may play important roles in strengthening the formation and function of soft feces.

## 5. Conclusions

In summary, our study demonstrated that microbial composition and function differed greatly between fecal types, and that soft feces harbored more microbes associated with active metabolism of energy, vitamins, and amino acids. These findings expand our knowledge regarding differential adaptation implications of gut microbiota between fecal types, as well as the links between gut microbiota and cecotrophy in pikas.

## Data Availability

The 16S rDNA data generated in this study can be freely retrieved from the NCBI Sequence Read Archive with project accession no. PRJNA609404 (https://dataview.ncbi.nlm.nih.gov/object/PRJNA609404?reviewer=ua6pp4jtollj23rpvhk5uleo1l).

## Supplementary Materials

The following supporting information can be downloaded at: https://www.mdpi.com/article/10.3390/ani12020149/s1: Figure S1. (a) The relative abundance of core microbiota at phylum level in hard and soft feces. (b) The relative abundance of core microbiota at genus level in hard and soft feces. (c) The linear discriminant analysis effective size (LEfSe) analysis shows the different taxa of core microbiota between hard and soft feces (*p* < 0.05, LDA scores > 2.0); Figure S2. Enriched KEGG map in Figure 5b, where red square frames represent the upregulated genes (K-numbers). (a) Citrate cycle (carbohydrate metabolism), (b) oxidative phosphorylation (energy metabolism), (c) C5-branched dibasic acid metabolism (carbohydrate metabolism), (d) carbon metabolism (global and overview), and (e) carbon fixation pathways in prokaryotes (energy metabolism); Figure S3. Enriched KEGG map in Figure 5b, where red square frames represent the upregulated genes (K-numbers). (a) Lipopolysaccharide biosynthesis (glycan biosynthesis and metabolism), (b) sphingolipid metabolism (lipid metabolism), (c) lipoic acid metabolism (metabolism of cofactors and vitamins), and (d) other glycan degradation (glycan biosynthesis and metabolism); Table S1. Sample information of 16S rDNA sequencing data with hard and soft feces; Table S2. Relative abundance of gut microbiota in hard and soft feces at phylum level; Table S3. Relative abundance of gut microbiota in hard and soft feces at genus level.
