# Supplementary material for: Metagenomic Comparisons between Soft and Hard Feces of Plateau Pikas (Ochotona curzoniae)
Source: Animals (Basel). 2022 Jan 8;12(2):149. doi: 10.3390/ani12020149 (PMC8772556; doi:10.3390/ani12020149)
Supplement: Supplementary file 1 [file animals-12-00149-s001.zip › NEW - Supplementary Materials.docx]

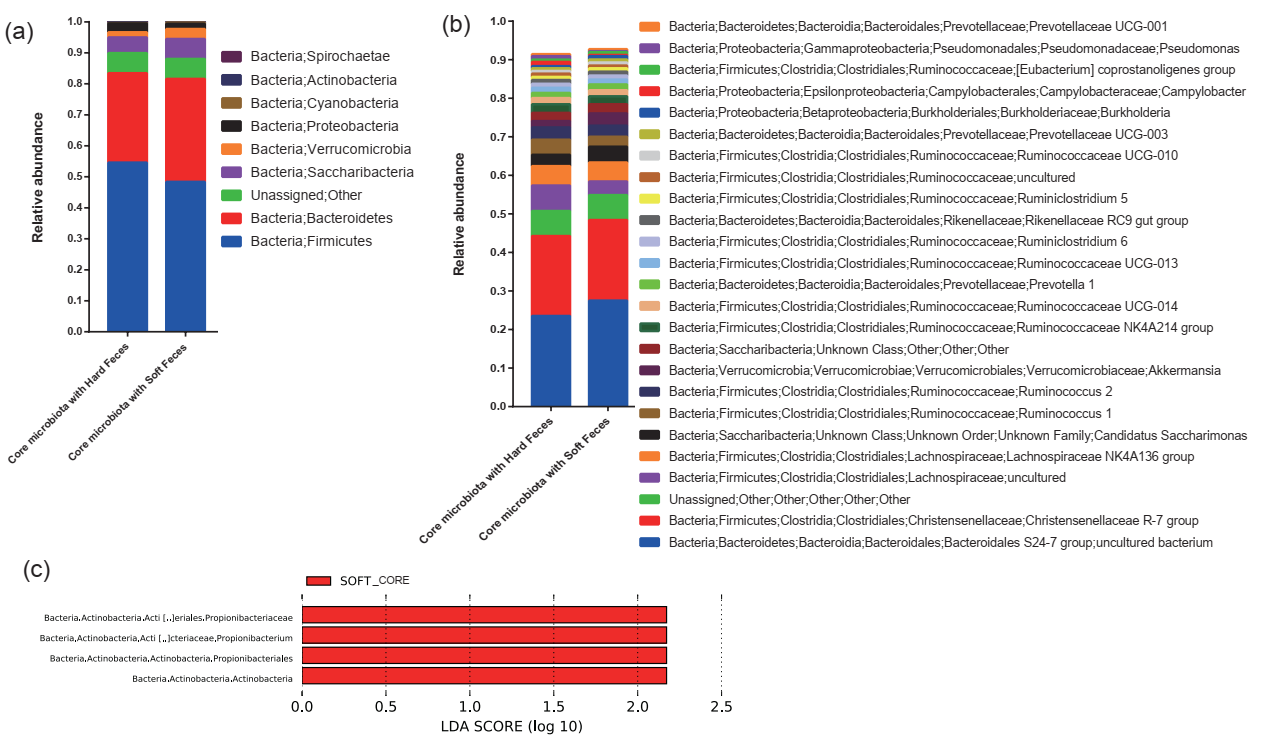


**Figure S1**. (a) The relative abundance of core microbiota at phylum level in hard and soft feces. (b) The relative abundance of core microbiota at genus level in hard and soft feces. (c) The linear discriminant analysis effective size (LEfSe) analysis shows the different taxa of core microbiota between hard and soft feces (*p* < 0.05, LDA scores > 2.0)


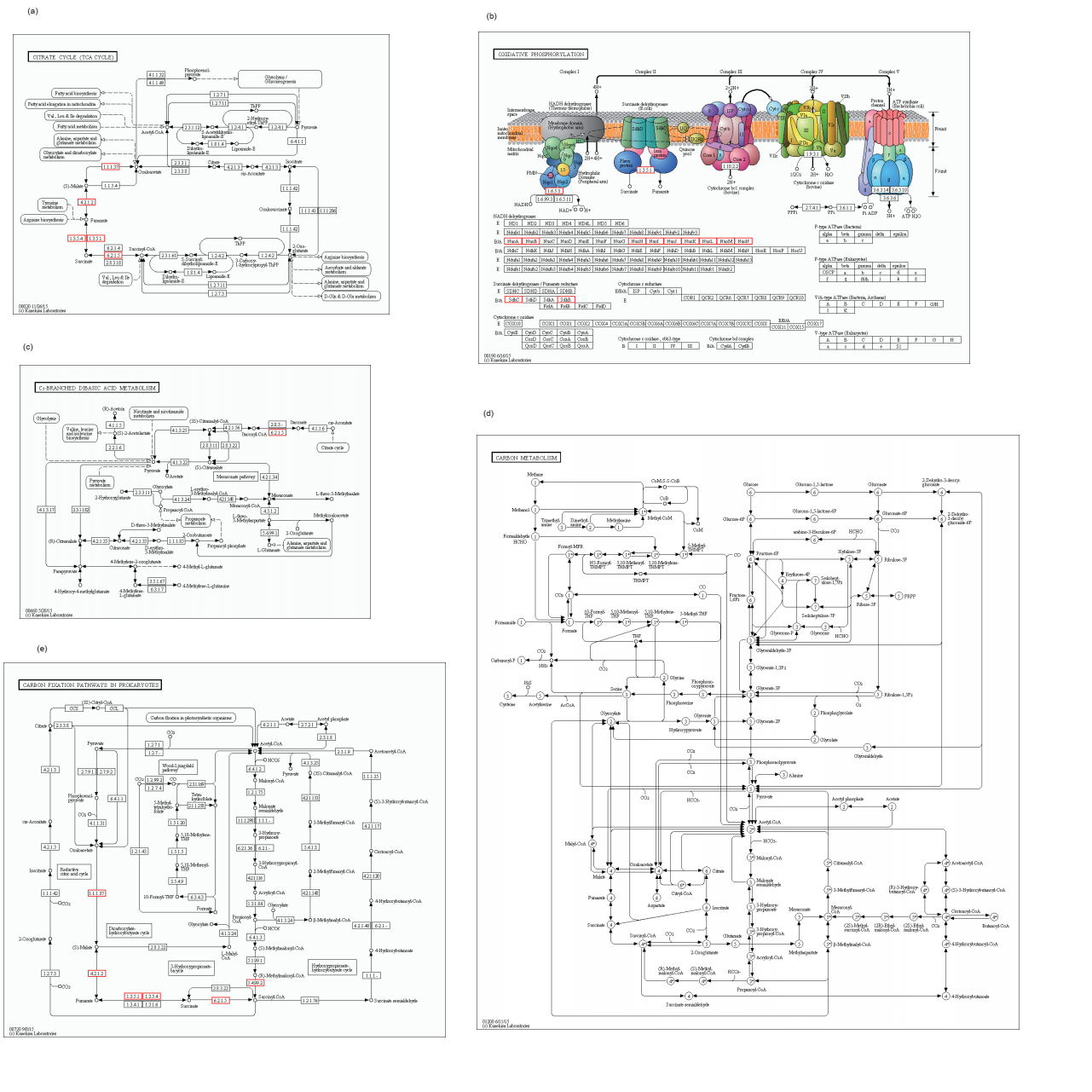


**Figure S2.** Enriched KEGG map in Figure 5b, where red square frames represent the upregulated genes (K-numbers). (a) Citrate cycle (carbohydrate metabolism), (b) oxidative phosphorylation (energy metabolism), (c) C5-branched dibasic acid metabolism (carbohydrate metabolism), (d) carbon metabolism (global and overview), and (e) carbon fixation pathways in prokaryotes (energy metabolism)


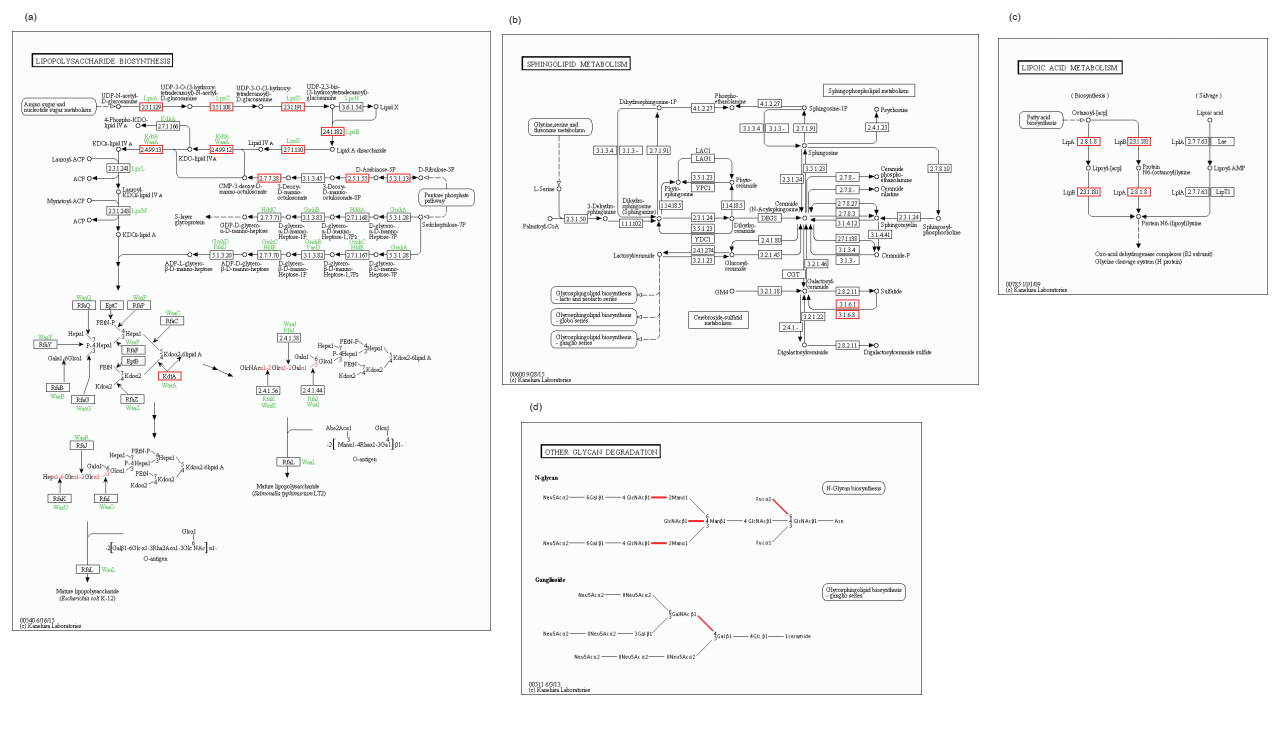


**Figure S3.** Enriched KEGG map in Figure 5b, where red square frames represent the upregulated genes (K-numbers). (a) Lipopolysaccharide biosynthesis (glycan biosynthesis and metabolism), (b) sphingolipid metabolism (lipid metabolism), (c) lipoic acid metabolism (metabolism of cofactors and vitamins), and (d) other glycan degradation (glycan biosynthesis and metabolism)

**Table S1.** Sample information of 16S rDNA sequencing data with hard and soft feces.

| **Sample number** | | **Age** | **Sex** | **Sample Type** | **Clean OTU sequence count** |
| --- | --- | --- | --- | --- | --- |
| H1 | Adult | | female | Hard Feces | 45,604 |
| H2 | Adult | | female | Hard Feces | 131,495 |
| H3 | Adult | | female | Hard Feces | 145,071 |
| H5 | Adult | | female | Hard Feces | 103,249 |
| H6 | Adult | | female | Hard Feces | 92,343 |
| H7 | Adult | | male | Hard Feces | 293,795 |
| H8 | Adult | | male | Hard Feces | 70,037 |
| H9 | Adult | | female | Hard Feces | 68,464 |
| H10 | Adult | | male | Hard Feces | 45,882 |
| H12 | Adult | | female | Hard Feces | 90,959 |
| S1 | Adult | | female | Soft Feces | 117,664 |
| S2 | Adult | | female | Soft Feces | 108,972 |
| S3 | Adult | | female | Soft Feces | 77,660 |
| S5 | Adult | | female | Soft Feces | 131,735 |
| S6 | Adult | | female | Soft Feces | 85,955 |
| S7 | Adult | | male | Soft Feces | 88,566 |
| S8 | Adult | | male | Soft Feces | 68,336 |
| S9 | Adult | | female | Soft Feces | 53,923 |
| S10 | Adult | | male | Soft Feces | 79,194 |
| S12 | Adult | | female | Soft Feces | 94,503 |
|  |  | |  |  | Total count: 2,529,464 |
